# The efficacy of acetic acid and citric acid on calcium silicate based cement removal from root canals and their effect on dentine structure – an *in vitro* study

**DOI:** 10.2340/biid.v13.45669

**Published:** 2026-03-25

**Authors:** Hanan Alzraikat, Ahmad S. Al-Hiyasat, Estabraq Sarya Jamous, Mohammad A. Alebrahim

**Affiliations:** a Department of Conservative Dentistry, Faculty of Dentistry, Jordan University of Science & Technology, Irbid, Jordan; b Department of Allied Medical Sciences, Faculty of Applied Medical Sciences, Jordan University of Science & Technology, Irbid, Jordan

**Keywords:** microhardness, tricalcium silicates, calcium silicate cements retrieval, acid solvents, acetic acid, citric acid

## Abstract

**Objectives:**

This study evaluated the effectiveness of acids on calcium silicate cement surface hardness, perforation, and their impact on root dentine microstructure.

**Materials and methods:**

Root dentine discs were exposed to hydrochloric, acetic, and citric acids. Fourier transform infrared spectroscopy (FTIR) assessed dentine microstructure after exposure to these acids. Sixty discs of Biodentine and NeoPUTTY (*n* = 30 each) were treated with 2% acetic acid, 5% citric acid, or saline (control), then tested for surface microhardness (Vickers hardness number, VHN). Another 60 dentine discs filled with these cements were exposed to the acids or saline and mechanically perforated with a Peeso reamer; perforation time was recorded. Scanning electron microscopy (SEM) examined dentine and cement surfaces.

**Results:**

FTIR revealed that hydrochloric acid caused severe demineralization of dentine microstructure, whereas 2% acetic and 5% citric acids had no significant effect on dentine microstructure. Both acids (acetic and citric) significantly reduced VHN compared to controls (*p* < 0.001). Acid-treated groups were perforated faster, with citric acid showing the greatest time reduction (*p* < 0.001). NeoPUTTY required less perforation time than Biodentine (*p* < 0.001). SEM revealed surface cracks in Biodentine and round-shaped defects in NeoPUTTY after acid exposure; citric acid opened dentinal tubules more distinctly than acetic acid.

**Conclusions:**

Acetic and citric acids significantly reduced microhardness and facilitated mechanical perforation of calcium silicate cements, with citric acid exerting greater effect.

KEY MESSAGES

1. A 1-minute application of citric acid significantly reduced the microhardness and perforation time of calcium silicate cements; this finding suggests that short-term citric acid application may facilitate cement retrieval when removal is required.
2. NeoPUTTY required less time for removal than Biodentine, suggesting it may be more favorable in clinical scenarios where retreatment is anticipated.

## Introduction

Calcium silicate-based materials are self-setting hydraulic cements that have become integral in various endodontic treatments such as apexification or creating apical plugs, repairing root perforations, vital pulp therapy, and pulp revascularization [1]. Their widespread use is attributed to their unique ability to stimulate biomineralization promoting tissue regeneration and healing [2]. In addition, they are well known for their high biocompatibility, antibacterial efficiency, and good sealing ability enhancing the long-term success of treatments [3].

Despite the high success rate of root canal therapy [4] including regenerative treatment and vital pulp therapy, treatment failure may still occur in some clinical cases. These failures may result from increased material solubility over time [5, 6] or the failure of the initially planned treatment [7]. In such cases, retreatment becomes essential and requires the complete removal of any material from the pulp chamber and root canal to re-establish access to the apical foramen with as little change to the root canal’s anatomy as possible [8]. This is particularly relevant in regenerative endodontic procedures and apical plug techniques, where calcium silicate–based materials, typically supplied in a putty-type consistency, are applied in the coronal part of the root canal in the regenerative procedures or apical part as an apical plug [9, 10]. However, concerns have been raised regarding retreatment procedures in teeth where calcium silicate–based materials have been placed. These concerns are mainly due to the fact that complete removal of calcium silicates after setting is challenging as they set into a hard mass. Consequently, their retrievability becomes difficult and can pose significant procedural errors [11], especially in cases with compromised tooth structures or open apices [7].

Mechanical tools such as conventional rotary and ultrasonic instruments have been used in an attempt to successfully remove calcium silicate cements such as Mineral trioxide aggregate (MTA) but have proven to be ineffective [12]. Several studies have examined the effect of an acidic environment on calcium silicate materials, concluding that low pH values of acidic solutions can negatively impact their surface morphology [13], microhardness [14], push-out bond strength [15], and sealing ability [16]. In addition, numerous studies have evaluated the efficacy of various solvents in calcium silicate materials’ retrieval [17–22]. These solvents include hydrochloric acid, carbonic acid, acetic acid, citric acid, tartaric acid, glycolic acid, sodium hypochlorite (NaOCL), chlorhexidine, and ethylenediaminetetraacetic acid (EDTA).

Several solvents have shown limited efficacy in dissolving calcium silicate–based materials, while others, although they were effective, they have application times and methods that do not simulate clinical conditions or applications. For instance, some studies applied acids by immersing cement samples in specific solutions [18, 19], while other researchers have employed continuous irrigation methods [17]. The reported application times of solvents on calcium silicate-based cements vary widely across the studies. Exposure durations included 5, 10, 15, and 20 minutes [19], 15 minutes at a rate of one drop per minute [22], and 10 minutes of contact at specific time periods [23]. Shorter applications, such as 5 minutes [20] and 10 minutes of immersion [18], were likewise documented.

Despite the fact that those studies have investigated the use of acidic solutions for dissolving calcium silicate cements, their impact on root dentine remains insufficiently explored [19, 21, 22]. Dentine is composed of 70% mineral (inorganic) content, 20% organic matrix, and 10% water by weight with calcium (Ca) and phosphorus (P) being the main inorganic components [24]. Ideally, solvents should target only the cement without affecting the integrity of dentine as exposure to these acidic solvents may alter the ratio of organic to inorganic components of dentine, leading to changes in microhardness, increased permeability and solubility, and reduced resistance to bacterial ingress, ultimately resulting in leakage [25]. Some studies have examined dentine surface hardness in response to different solvents [17, 18, 23], however, there is insufficient evidence regarding the optimal acid or solvent concentration that can dissolve or facilitate the mechanical removal and retrieval of calcium silicate cements from root canals and the effect of these solvents on dentine microstructure and crystallinity.

In view of the aforesaid, this study aimed to determine the optimal acid type and concentration that facilitates removal of calcium silicate materials from root canals without adversely affecting root dentine structure.

## Materials and method

### Preliminary study

The research project was approved by the research committee at the university of the researcher. A pilot study was conducted to determine the optimal concentration and type of acidic solutions that could be used without significant effect on root dentine microstructure and crystallinity using Fourier transform infrared spectroscopy (FTIR). The pilot study was performed solely to optimize the experimental conditions, and no pilot data were included in the final analysis of the main study.

Twenty-five freshly extracted mature vital human single rooted premolars that were extracted for orthodontic reason were collected. The teeth were free of caries, resorption or cracks, and with intact crowns. Immediately after extraction, the selected teeth were cleaned using an ultrasonic scaler and stored in 0.1% Thymol solution (Alpha Chemika, Andheri, India). Root dentine discs (1.5–2 mm-thick) were obtained from the root of each tooth below the cemento-enamel junction (CEJ); 2–3 discs were taken from each tooth from coronal and middle thirds using a diamond saw (Buehler, Lake Bluff, IL, USA) under water irrigation. Discs were randomly divided into seven groups (*n* = 5/group) according to the applied solutions. Approximately 20 microliters of each solution were applied over each disc using a micropipette (Finnpipette F3, Thermo Fisher Scientific, Cleveland, OH, USA), which is equivalent to approximately three drops when using an endodontic irrigation tip. The solutions used, their concentrations, and pH values are shown in Table 1.

**Table 1:** Acids and control solution used in the study.

Group #	Acid concentration %	pH
**Group 1 (control)**	0.9% Normal saline	6.7
**Group 2**	25% HCl	0.45
**Group 3**	35% HCl	0.22
**Group 4**	2% Acetic acid	2.8
**Group 5**	4% Acetic acid	2.27
**Group 6**	5% Citric acid	2.6
**Group 7**	10% Citric acid	2.15

Manufacturer information for used acids:

(Hydrochloric acid, Rasayan Udyog, Kanpur, Uttar Pradesh, India)

(Citric acid anhydrous, Scharlau Chemie S.A. La Jota, Barcelona, Spain)

(Acetic acid, Sinopharm Chemical Reagent Co., Ltd., Shanghai, China)

After exposing the samples to the assigned solution for 1 minute at room temperature, all samples were then subjected to Attenuated Total Reflection-Fourier Transform Infrared spectroscopy (ATR-FTIR) (TENSOR II, platinum ATR, Bruker Optik GmbH). The percentage of spectra ranged from 400 to 4000 cm^−1^ and the spectrum of each sample was collected as an average of 32 scans at a resolution of 4 cm^−1^ [26]. Data were analyzed using Origin Pro 2020 software (OriginLab Corporation, Northampton, MA, USA).

The FTIR spectral analysis qualitatively focused on the phosphate region (900 –1150 cm^−1^), which reflects the mineral contents and crystallinity of the dentine. To further confirm these findings, the integrated area of the phosphate band at 960 cm^−1^ and its associated full width at half maximum (FWHM) were quantitatively assessed. A larger integrated area of the phosphate band typically indicates a denser, more mineralized tooth structure with a highly crystalline arrangement. In contrast, the FWHM values of the phosphate band is inversely related to mineral crystallinity – broader FWHM values suggest a lower degree of crystallinity in the tooth structure.

### Microhardness and perforation tests of the cement samples

#### Microhardness

The sample size for each group was determined based on effect sizes reported in the literature [19, 21]. A priori power analysis was performed using G*Power 3.1, with a significance level of α = 0.05 and a power of 0.80, which indicated that 10 specimens per group were required. Sixty hollow cylindrical plexiglass molds (2 mm height × 5 mm internal diameter) were fabricated and divided into two main groups (*n* = 30) according to the tested material: group 1 Biodentine (Septodont, Saint Maur-des-Fossés Cedex, France) and group 2 NeoPUTTY (Avalon Biomed Inc., Bradenton, USA). The composition of each material is shown in Table 2.

**Table 2:** Biodentine and NeoPUTTY composition.

Materials	Composition
**Biodentine**	Powder: Tricalcium silicate (Ca_3_SiO_5_), dicalcium silicate (Ca_2_SiO_4_), calcium carbonate (CaCO_3_), iron oxide (Fe_2_O_3_), and zirconium oxide (ZrO_2_) Liquid: Water (H_2_O) with calcium chloride (CaCl_2_) and soluble polymer (polycarboxylate)
**NeoPUTTY**	Tricalcium silicate, dicalcium silicate powder, tantalum oxide (tantalite) in an anhydrous organic liquid

Molds were filled to excess with freshly prepared cement. Excess material was trimmed off to the level of the mold surface using a blade, then wet cotton pellets were applied on NeoPUTTY samples to ensure proper setting as recommended by the manufacturer. All samples were stored in an incubator at 37°C and 100% humidity for 3 days to allow complete setting. Subsequently, all the samples were examined with a microscope and any samples with cracks, defects, or gaps between the material and the mold was discarded. Both sides of each sample in each group were then polished using Shofu super-snap polishing disks (Shofu Inc., Kyoto, Japan) under water cooling.

Each group was then subdivided into three groups (*n* = 10) according to the solution applied as follows: subgroup 1 was exposed to 2% acetic acid, subgroup 2–5% citric acid, and subgroup 3 to normal saline (control group). Samples were exposed to the solution to completely cover the cement surface sample by applying approximately 3 drops of the assigned solution for 1 minute, then rinsed with distilled water for 1 minute, and dried using kimwipes (Kimtech; Kimberly-Clark, TX, USA). The polished surface prepared prior to acid solution application was not re-polished after exposure, as the aim was to evaluate the direct effect of the acid solutions on surface microhardness. This ensured that the tested surface represented the true material changes induced by the acidic environment. Hardness measurements were performed using Vickers microhardness testing machine (MHV 2000Z, SCTMC, Shanghai China). Three indentations were made on the polished surface of each sample using a load of 100 g for 15 seconds of dwell time at room temperature. The Vickers hardness number (VHN) was calculated for each indentation using the formula (VHN = 1.854(F/D^2^)), F refers to the applied force in kilograms and D refers to the mean diagonal length of the indentation in millimeters. Then the mean value of the three indentations was calculated as the hardness value for each sample.

#### Perforation test

The perforation test model was designed to simulate the confined intracanal environment encountered during retreatment of regenerative or apical plug procedures. Dentine discs were selected to replicate a natural canal substrate, and Peeso reamers were used because they allow standardized vertical penetration within a narrow canal-like space.

Sixty single rooted premolars (selected according to the criteria mentioned previously) that were freshly extracted for orthodontic reasons were cleaned using an ultrasonic scaler and stored in 0.1% Thymol. The working length of each canal was established with a size 10 K-file (Dentsply Maillefer, Ballaigues, Switzerland) introduced into each root canal until its tip was visualized at the apex and then pulled back 1 mm. The root canal of each tooth was then prepared using Protaper Gold system up to F3 (Dentsply Maillefer, Ballaigues, Switzerland). Root canal irrigation during preparation and instrumentation was done using 5% NaOCL followed by irrigation with 17% EDTA for 1 minute to remove the smear layer, then a final rinse with distilled water was performed. Each root canal was then enlarged with Gates Glidden size 1,2,3 to the full length (Dentsply Maillefer, Ballaigues, Switzerland) in order to widen the canal lumen [23] then each canal was washed with distilled water. Subsequently, the root of each tooth was imbedded into self-cure acrylic resin (Ece Boya Kimya, Istanbul, Turkey) in a cylindrical silicon mold. A 4 mm thick disc was sectioned from the coronal to middle thirds of each root. The discs were then divided randomly into two main groups (*n* = 30) according to the tested cement, group 1 Biodentine and group 2 NeoPUTTY. Root canal sections were filled with cement accordingly using a hand plugger, and excess material was removed by a blade to ensure a flat surface. A cotton pellet moistened with water was placed on each sample filled with NeoPUTTY to facilitate its setting (as recommended by the manufacturer). The samples were stored for 3 days in an incubator at 37°C and 100% humidity following manufacturer instructions to allow complete setting.

**Figure 1:**
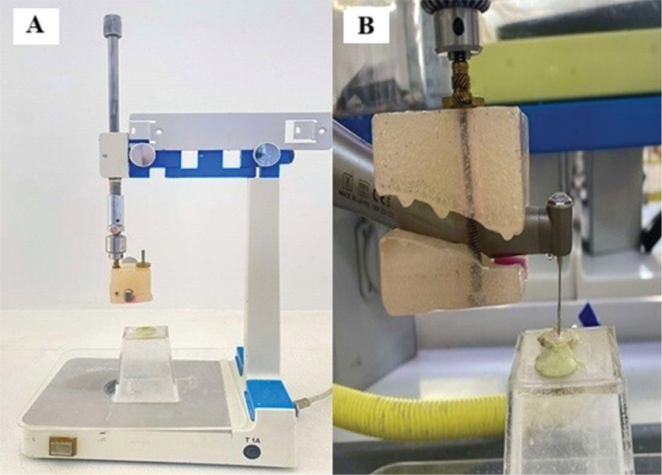
(A) Surveyor with the assembly of handpiece and holder, (B) Sample subjected to mechanical perforation test.

Each main group was further subdivided into three groups (*n* = 10) then each group was subjected to the assigned acidic solution (2% acetic acid, 5% citric acid, and normal saline as a control as mentioned before), rinsed, and dried as described in the previous section. Then, the Biodentine and NeoPUTTY samples filled in the dentine discs were subjected to a drilling force using Peeso reamer (size 3) mounted on a slow-speed handpiece operating at 40,000 rpm (NSK, Nakanichi Inc., Tochigi, Japan). A new Peeso reamer was used after every two samples to minimize instrument fatigue. The handpiece was secured onto a surveyor device (Degussa, Geschäftsbereich Dental, Frankfurt, Germany) using a custom-fabricated acrylic resin holder (Figure 1A). This assembly, comprising the handpiece and holder, was mounted in a manner that allowed vertical movement facilitated by a spring mechanism. Although absolute force values were not measured, the spring-loaded surveyor allowed the handpiece to descend from a fixed height, ensuring consistent relative pressure across all samples. Consequently, during the mechanical perforation test, the assembly descended uniformly for every specimen, providing comparable downward pressure of the Peeso reamer on all samples. A specialized holder with a central opening was designed to enable precise positioning of the handpiece over the root canal space of each sample, which was prefilled with the tested cement. Additionally, samples were securely fixed in position using an adhesive material (Figure 1B). The time required to completely perforate the Biodentine and NeoPUTTY samples (that was ensured with the sudden drop of the handpiece) was measured in seconds using a stopwatch.

#### Scanning electron microscopy

Twelve root canal discs were prepared as described previously and were randomly assigned to two main groups six-discs each: Group 1 Biodentine and Group 2 NeoPUTTY. The root canal sections were filled accordingly either with Biodentine or NeoPUTTY as described before, and allowed to set as instructed by the manufacturer. After complete setting, samples in each group were polished with 600-grit,1200-grit, and 2000-grit silicon carbide papers (3M, St Paul, MN, USA) under water cooling to create a flat and clean surface of each sample to produce clear images by scanning electron microscopy (SEM). Then each group was further subdivided into three subgroups (*n* = 2), subgroup 1 was exposed to 2% acetic acid, subgroup 2–5% citric acid, and subgroup 3 to normal saline. Then samples were exposed to solutions and rinsed with distilled water then dried as described previously. Samples were mounted on aluminum stubs, sputter coated with gold (Q150R ES sputter coater, Quorum Technologies, United Kingdom) and examined under SEM (Quanta FEG 450, FEI, Netherlands). Images were captured at 400×, 1300×, 2500×, 5000× and 10,000× magnifications to assess surface microstructural changes of dentine and tested cements.

### Statistical analysis

Means and standard deviations were measured for the tested groups. Shapiro–Wilk and Kolmogorov–Smirnov tests were used to test the normality of the data and residuals, and showed parametric distribution. Study data were statistically analyzed in terms of calcium silicate cement type and solutions (acids and control) as the two variables of the study using two-way (ANOVA) test. Post hoc and intergroup comparisons were conducted using the Bonferroni test. The level of significance was set at (α = 0.05) for all tests. For data analysis IBM SPSS statistics software (Version 29) was used.

## Results

### Microstructure and crystallinity of dentine – FTIR

**Figure 2:**
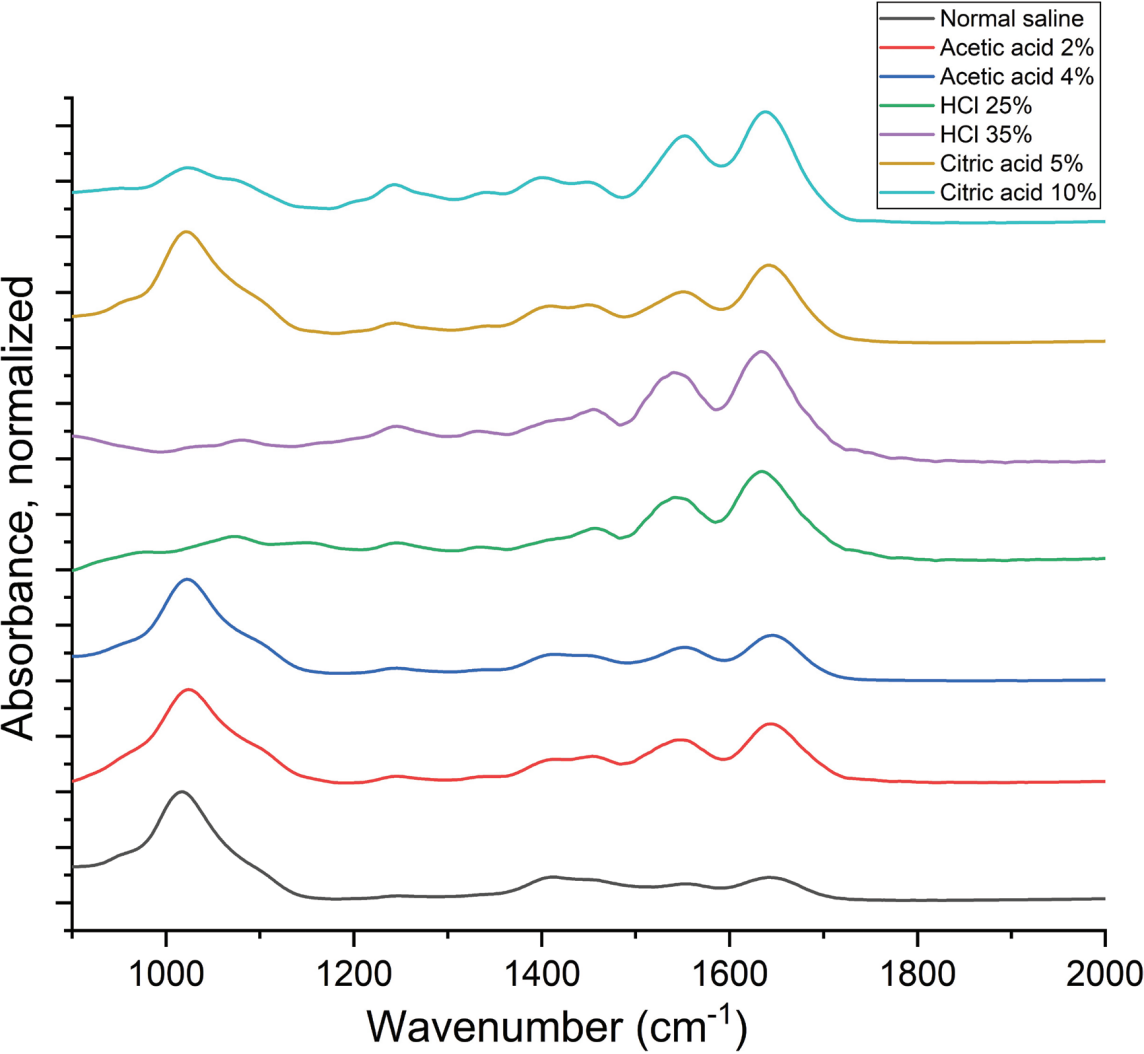
Infrared (IR) spectra of dentine Samples (900–2000 cm^−1^) after exposure to control and acidic solutions.

The FTIR data demonstrate the stability of dentine mineral in neutral environments, as represented by normal saline, which preserves the phosphate structure and crystallinity. Exposure to 25 and 35% HCl completely obliterated the phosphate profile (960 cm^−1^), reflecting near-total dissolution of the dentine’s mineral phase (Figure 2). After 1-minute exposure to 2 and 4% acetic acid, the 960 cm^−1^ band showed reduced area, suggesting partial demineralization and reduced mineral crystallinity. The effect was more pronounced with 4% acetic acid, where the 960 cm^−1^ band area dropped, indicating substantial mineral loss (Table 3). Teeth exposed to 5% citric acid had band heights similar to the control group, whereas 10% citric acid caused notable changes in band height, suggesting a concentration-dependent impact on the phosphate profile and mineral content. This is evident by the broadening of the 960 cm^−1^ band and reduction in its area.

**Table 3:** Integrated area of phosphate band and its full width at half maximum of tested solutions.

Solution	Area of 960 cm^–1^	960 cm^–1^ FWHM
Normal saline	9.53 ± 0.37^a^	64.13 ± 0.85^a^
HCl 25%	ND	ND
HCl 35%	ND	ND
Acetic acid 2%	6.23 ± 1.28^b^	91.23 ± 1.66^b^
Acetic acid 4%	4.17 ± 0.46^b^	93.68 ± 0.11^b^
Citric acid 5%	8.68 ± 0.52^a^	66.85 ± 1.11^a^
Citric acid 10%	6.99 ± 0.51^a,b^	72.58 ± 1.26^a,b^
*P*-value	< 0.001	< 0.001

ND = Not determined due to complete destruction of the phosphate profile following mineral dissolution.

FWHM: full width at half maximum.

Means followed by different lowercase letters (a, b) within the same column are significantly different (p < 0.05).

Therefore, based on the previous results, 5% citric acid and 2% acetic acid were selected for conducting the remaining tests in this study with a 1-minute exposure time in addition to normal saline as the control solution.

### Microhardness test

Mean VHN ± standard deviation of the tested cements after exposure to the acids and control (normal saline) were calculated and are shown in Table 4.

**Table 4:** Microhardness mean VHN ± SD for Biodentine and NeoPUTTY after exposure to the tested solutions.

Solution	Cement type		*P*-value
	Biodentine	NeoPUTTY	
**Normal saline**	83.02 ± 4.02 ^A,a^	41.30 ± 3.50 ^A,b^	< 0.001
**Acetic acid 2%**	71.28 ± 6.84 ^B,a^	31.26 ± 3.11 ^B,b^	< 0.001
**Citric acid 5%**	46.66 ± 3.97 ^C,a^	21.09 ± 1.99 ^C,b^	< 0.001
***P*-value**	< 0.001	< 0.001	

All values are expressed as mean ± SD. VHN: Vickers hardness number.

Means followed by different uppercase letters (A, B, C) within the same column indicate significant differences between solutions within the same cement group (*p* < 0.001).

Means followed by different lowercase letters (a, b) within the same row indicate significant differences between cement types within the same solution group (*p* < 0.001).

The two-way ANOVA revealed that both the type of material and the type of solution had a significant effect on Vickers microhardness values as well as the interaction between materials and solutions (*p* < 0.001). Post hoc pairwise comparisons showed that Biodentine exhibited higher VHN values than NeoPUTTY (*p* < 0.001) in all groups. Both acetic acid and citric acid significantly decreased the VHN values compared with saline for both materials (*p* < 0.001) with citric acid producing a significantly greater reduction in hardness values than acetic acid as shown in Table 4.

### Mechanical perforation test

Mean values ± standard deviation of the time needed to perforate Biodentine and NeoPUTTY samples were calculated and are shown in Table 5. Two-way analysis demonstrated that both cement type and solution had a significant effect on perforation time, with a significant interaction between these factors (*p* < 0.001). Pairwise comparisons showed that mechanical perforation of NeoPUTTY was significantly faster than Biodentine in all solution groups. The time needed to perforate both cements in the tested acid groups (acetic acid and citric acid) was significantly shorter than the control group (normal saline). Furthermore, Biodentine and NeoPUTTY were mechanically perforated at a significantly shorter amount of time in citric acid than acetic acid.

**Table 5:** Mean time ± SD (seconds) needed to perforate tested cements.

Solution	Cement type		*P*-value
	Biodentine	NeoPUTTY	
**Normal saline**	110.98 ± 2.75 ^A,a^	40.46 ± 2.37 ^A,b^	< 0.001
**Acetic acid 2%**	96.25 ± 2.04 ^B,a^	25.69 ± 3.00 ^B,b^	< 0.001
**Citric acid 5%**	72.94 ± 2.71 ^C,a^	12.84 ± 1.78 ^C,b^	< 0.001
***P*-value**	< 0.001	< 0.001	

All values are expressed as mean ± SD.

Means followed by different uppercase letters (A, B, C) within the same column indicate significant differences between solutions within the same cement group (*p* < 0.001).

Means followed by different lowercase letters (a, b) within the same row indicate significant differences between cement types within the same solution group (*p* < 0.001).

### SEM observations

**Figure 3:**
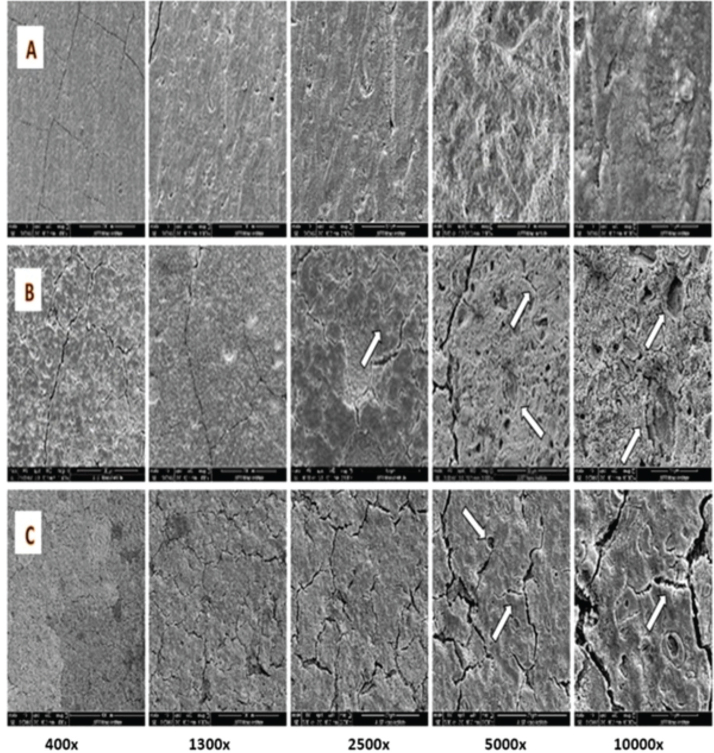
SEM of Biodentine surface, (A) after exposure to normal saline, (B) after exposure to 5% citric acid, (C) after exposure to 2% acetic acid. Arrows point to porosities and micro-channels. SEM: scanning electron microscopy.

**Figure 4:**
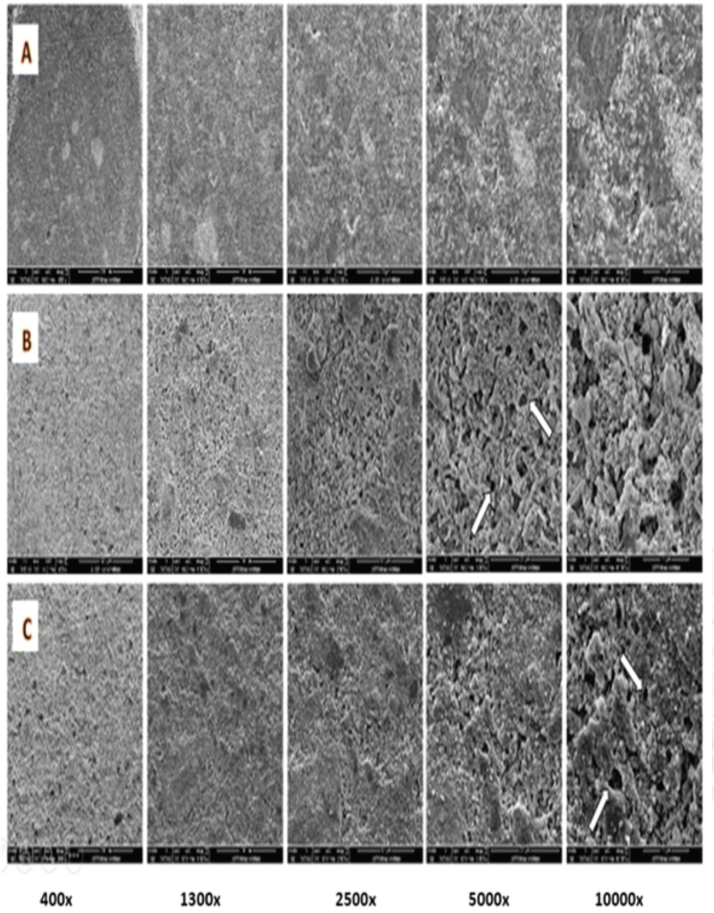
SEM of NeoPUTTY surface, (A) after exposure to normal saline, (B) after exposure to 5% citric acid, (C) after exposure to 2% acetic acid, arrows pointed at hollow pits. SEM: scanning electron microscopy.

The surfaces of Biodentine and NeoPUTTY samples after exposure to acids and control solution are shown in Figure 3 and Figure 4, respectively. Five magnifications were used, namely 400×, 1300×, 2500×, 5000× and 10,000×. Biodentine samples showed surface cracks after exposure to both acetic acid and citric acid compared to the control group (Figure 3). The surface of Biodentine subjected to 5% citric acid in Figure 3B demonstrated cement matrix destruction, micro-channels, and porosities at 2500× and 5000× magnifications (as indicated by arrows). Comparable observations were seen in the 2% acetic acid group sample (Figure 3C). However, at a higher magnification there is a coarser and more irregular structure in the citric acid group sample.

The NeoPUTTY samples subjected to normal saline exhibited a uniform and homogenous surface texture. In contrast, the samples exposed to the acids showed significant surface degradation characterized by numerous round-shaped defects and hollow pits (Figure 4, as indicated by arrows). Notably, the citric acid group exhibited more pronounced defects and greater matrix loss compared to the acetic acid group, indicating a higher degree of material breakdown.

**Figure 5:**
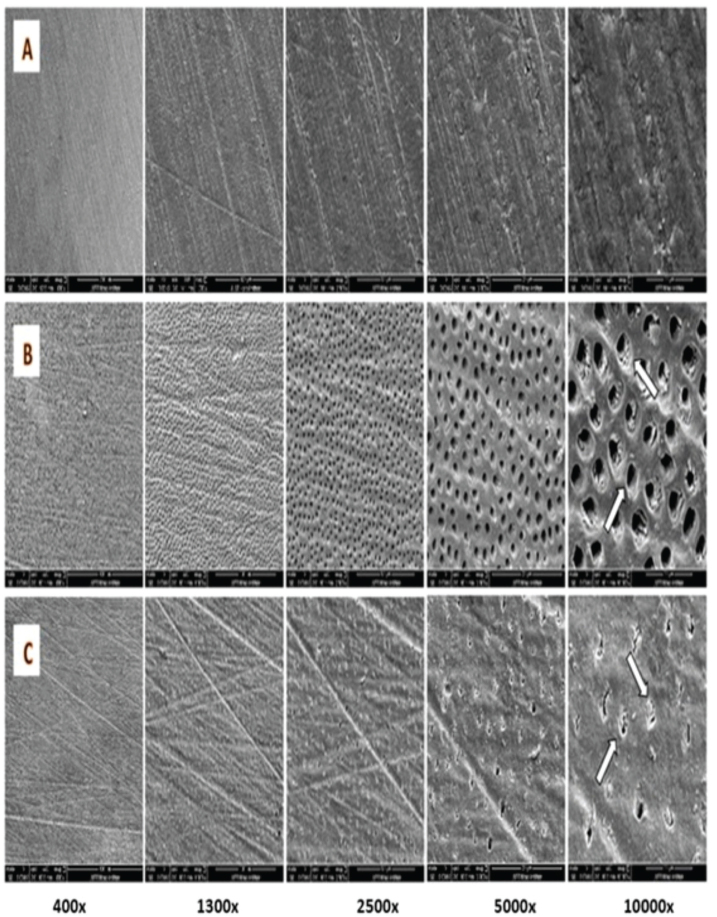
SEM of root dentine surface, (A) after exposure to normal saline, (B) after exposure to 5% citric acid, (C) after exposure to 2% acetic acid, arrows pointed at dentinal tubules. SEM: scanning electron microscopy.

Root dentine samples surrounding the tested cements in each sample were examined after exposure to normal saline and acid solutions and are shown in Figure 5. Saline-treated dentine showed no surface changes (Figure 5A). While surface changes of root dentine were observed in citric acid and acetic acid treated samples (Figure 5B and C). Dentinal tubular orifices were clear and open in samples subjected to 5% citric acid as indicated by the arrows in Figure 5B at 10,000× magnification. Whereas in the acetic acid treated dentine, erosion with slightly widened dentinal tubules and remnants of the intratubular material and debris are detected as indicated by the arrows in Figure 5C.

## Discussion

In this study, the effectiveness of different acids in reducing the surface hardness of calcium silicate materials to facilitate their removal was evaluated using microhardness and mechanical perforation tests. Several studies have investigated the effect of a number of acidic solutions and solvents on the microhardness of calcium silicate cements [17–19, 21]. However, their impact on root dentine microstructure and crystallinity has not been properly explored. Therefore, the analysis of the this study samples focused on calculating the integrated area of the phosphate profile ranging at 960 cm^−1^ and determine the FWHM in order to determine the impact of those used acids on dentine crystallinity after 1-minute application time.

The FTIR results of the pilot study showed that the phosphate profile, spanning the range of 900–1150 cm^−1^ [27], had been completely obliterated as a consequence of exposure to HCl. Contrary to these results, 2% acetic acid did not have a notable adverse effect on the phosphate profile when compared to the control samples. This result can be attributed to the fact that HCl is a strong mineral acid [28], and it exerts a significantly more corrosive effect on the samples, leading to the elimination of the phosphate bands. On the other hand, acetic acid, a weaker organic acid, does not manifest the same corrosive impact, hence the absence of marked changes in the spectral profile in comparison to the control samples as confirmed by Alebrahim et al. [26]. In this study, a significant difference in phosphate band at of 960 cm^−1^ in tooth samples exposed to 10% citric acid was observed, which is not seen when exposing dentine to 5% citric acid. This spectral signature suggests a distinct effect on teeth phosphate composition at higher citric acid concentrations. This is in agreement with another study, which showed that increasing the acid’s concentration increased the erosion of dentine and enamel [29].

In this study, 2–3 drops of each acid were applied on the surface of prepared samples in contrast to some studies that utilized the method of immersion of tested cement materials in acid solutions [18]. However, in a clinical setting, only one surface of the cements can be exposed to the solutions. In addition to measuring surface microhardness of tested materials, measuring the perforation time provides an indirect estimate of the acid’s effectiveness in softening or weakening the tested cements, which in turn could reduce the clinical working time required during subsequent removal.

The results of the microhardness measurements showed that both acetic and citric acids caused a significant reduction in the microhardness of both Biodentine and NeoPUTTY, with 5% citric acid yielding the greatest reduction. These findings align with a study by Halder et al. [18], which demonstrated that citric acid reduced the surface microhardness of Biodentine more significantly than acetic and tartaric acids. This can be attributed to citric acid’s calcium-depleting properties and its ability to interact with the mineral phase of the cements, which ultimately reduces the amount of di- and tri-calcium silicate phases [23]. The low pH value of 2.6 can also weaken calcium silicate-based materials by disrupting the crystallization of calcium silicate-hydrate (C-S-H) [17]. Previous studies examining the effect of citric acid on the calcium silicate cements hardness have also shown similar results when applying 10% citric acid for a range of time periods (5–20 minutes) [17, 18, 20]. In contrast, this study utilized a lower citric acid concentration and a shorter application time of 1 minute demonstrating the effectiveness of this acid in reducing cement’s hardness even in lower concentrations and a shorter application time.

The application of acetic acid also caused a significant reduction in microhardness of both cements in this study, a finding supported by other investigators [18, 23]. This effect is also attributed to acetic acid’s calcium-depleting action and its carboxyl group, which inhibits the crystalline formation of calcium hydroxide in Biodentine and the formation of calcium tartrate hydrate, which in turn reduces the amounts of tri- and di-calcium silicate and tricalcium aluminate phases [23, 30]. These chemical interactions suggest that organic acids (such as citric and acetic acids) may be effective in dissolving calcium silicate cements during retreatment procedures.

The results of the mechanical perforation test revealed that NeoPUTTY was mechanically perforated at a faster rate compared to Biodentine. The exposure of NeoPUTTY to acetic and citric acid solutions decreased the time needed to perforate the cement from 40 seconds (normal saline group) to 25 and 12 seconds, respectively. Similarly, Biodentine samples showed a decrease in the perforation time when subjected to acetic and citric acids compared to control samples. There was a 14-second decrease in perforation time between the control samples and acetic acid, while mechanical perforation of Biodentine subjected to citric acid decreased by 40 seconds. This can be attributed to the fact that when citric acid interacted with calcium silicate it led to the formation of a hydrosoluble chelate, which is easier to remove mechanically [31].

Overall, Biodentine required a significantly longer time to be perforated than NeoPUTTY in all solutions, indicating that Biodentine retrieval may be more challenging under the test conditions. This observation should be interpreted cautiously, as differences in internal microstructure and material disintegration behavior during drilling may also influence perforation time. The longer perforation time observed for Biodentine may be explained by its higher mechanical properties, including higher flexural strength, elastic modulus and Vickers hardness, which have been reported to be higher than those of other calcium silicate cements, including MTA [32]. Furthermore, Biodentine is reported to be denser and less porous when compared to MTA, which can be attributed to the differences in their chemical compositions, as Biodentine contains calcium carbonate, which forms a denser crystalline structure, enhancing its mechanical strength and hardness [33]. In contrast, MTA lacks calcium carbonate in its composition, leading to a more porous structure that can result in relatively lower mechanical strength and hardness with lower resistance to mechanical penetration and faster perforation than Biodentine. Additionally, Biodentine includes zirconium oxide as a radiopacifier, which is known for its favorable mechanical properties and resistance to corrosion [34]. Within this context, it is important to interpret the perforation findings in relation to the specific mechanical model used in this study. Although Peeso reamers have a non-cutting, safe-ended tip, cement perforation occurred through lateral cutting and abrasion by the flutes during axial rotation, while the blunt tip guided the instrument along the canal axis and minimized lateral deviation [35]. Consequently, the observed differences reflect material performance under the standardized Peeso reamer-based perforation model, and other removal systems may interact differently with the materials and produce different outcomes.

SEM observations revealed significant damage to Biodentine samples exposed to both acids, including cracks, destruction of the cement matrix, micro-channels, and porosities. However, exposure to citric acid resulted in a higher level of porosity compared to acetic acid. This can be attributed to the acids’ interaction with the set cement matrix of Biodentine, which dissolves the matrix and releases calcium and aluminum salts in crystalline forms, leaving surface irregularities. These changes and a reduction in mechanical strength enhance Biodentine retrievability [18]. Exposure of NeoPUTTY to both citric and acetic acids led to the formation of round-shaped defects and hollow pits, which is consistent with findings by Lee et al. and Namzikhah et al. [14, 36] who reported that low pH exposure caused increased porosity in MTA. SEM analysis of the dentine surface revealed that while the smear layer covered the dentinal tubules in the saline control group, exposure to citric acid and acetic acid resulted in smear layer removal. In the citric acid group, dentinal tubules were clearly visible and open. In contrast, the acetic acid-treated dentine exhibited slightly widened tubules, with remnants of intratubular material and debris. This difference can be attributed to the stronger acidic nature of citric acid, which has a lower pKa for its first dissociation step and multiple carboxyl groups, making it more effective at proton donation in solution [14, 37]. As a result, citric acid produced a stronger chelating effect, leading to more pronounced widening of the dentinal tubules and increased porosity and cracking in calcium silicate cements exposed to citric acid.

This study has several limitations. Firstly, the experimental setup did not fully replicate the complex and dynamic environment of the human oral cavity. Secondly, the use of Peeso reamers for perforating the tested cements carries inherent risks to the underlying tooth structure. Since Peeso reamers are primarily designed for root canal preparation, their application in retrieving calcium silicate–based materials may generate excessive stress or unintended damage to the tooth. In this study, Peeso reamers in conjunction with dentine discs were used to simulate intracanal perforation under standardized conditions. However, alternative removal techniques using another type of rotary instruments that could be used to remove the calcium silicate cement from the root canal space may produce different outcomes and should be explored in future studies. Thirdly, retrieval or mechanical perforation was assessed after storing the tested cements in an incubator for 3 days to allow proper setting. However, the ability to remove these cements after longer storage periods warrants further investigation, as their mechanical properties continue to develop over time.

Future research should explore the mechanical removal of calcium silicate–based materials using rotary files or ultrasonic devices, with standardized pressure and orientation to ensure reproducibility. In addition, evaluating the retrieval of these materials from intact teeth – without sectioning – would more closely simulate clinical conditions. Expanding investigations to include other calcium silicate-based materials and bioceramic sealers is also recommended to broaden the scope and applicability of the findings. Additionally, investigating lower concentrations of HCl may be worthwhile given its well-known ability to dissolve mineralized substrates. Finally, clinical studies are needed to confirm the effectiveness of these acids, as their current support is limited to laboratory-based evidence of safety and efficacy.

## Conclusion

Within the limitations of this study, a 1-minute application of 5% citric acid significantly reduced the surface microhardness of the tested calcium silicate cements and shortened their perforation time. In addition, NeoPUTTY required significantly less time to be perforated than Biodentine, indicating that its retrieval may be less challenging under the tested conditions. These findings suggest that short-term application of citric acid may facilitate removal of calcium silicate materials when retreatment is required.

## Data Availability

The data that support the findings of this study are available from the corresponding author upon reasonable request.
